# Does It Count? Pre-School Children’s Spontaneous Focusing on Numerosity and Their Development of Arithmetical Skills at School

**DOI:** 10.3390/brainsci12030313

**Published:** 2022-02-25

**Authors:** Nadine Poltz, Sabine Quandte, Juliane Kohn, Karin Kucian, Anne Wyschkon, Michael von Aster, Günter Esser

**Affiliations:** 1 Department of Inclusive Education, University of Potsdam, 14476 Potsdam, Germany; 2 Academy of Psychotherapy and Intervention Research Potsdam, 14467 Potsdam, Germany; sabine.quandte@api-potsdam.de (S.Q.); juliane.kohn@api-potsdam.de (J.K.); anne.wyschkon@api-potsdam.de (A.W.); gesser@uni-potsdam.de (G.E.); 3 Center for MR-Research, University Children’s Hospital, 8032 Zurich, Switzerland; karin.kucian@kispi.uzh.ch; 4 Center for Special Educational and Psychological Needs, DRK Kliniken Berlin Westend, 14050 Berlin, Germany; m.aster@drk-kliniken-westend.de

**Keywords:** SFON, school mathematics, mathematical precursor, counting, number knowledge, magnitude estimation, transformation, pre-school, longitudinal, development

## Abstract

Background: Children’s spontaneous focusing on numerosity (SFON) is related to numerical skills. This study aimed to examine (1) the developmental trajectory of SFON and (2) the interrelations between SFON and early numerical skills at pre-school as well as their influence on arithmetical skills at school. Method: Overall, 1868 German pre-school children were repeatedly assessed until second grade. Nonverbal intelligence, visual attention, visuospatial working memory, SFON and numerical skills were assessed at age five (*M* = 63 months, Time 1) and age six (*M* = 72 months, Time 2), and arithmetic was assessed at second grade (*M* = 95 months, Time 3). Results: SFON increased significantly during pre-school. Path analyses revealed interrelations between SFON and several numerical skills, except number knowledge. Magnitude estimation and basic calculation skills (Time 1 and Time 2), and to a small degree number knowledge (Time 2), contributed directly to arithmetic in second grade. The connection between SFON and arithmetic was fully mediated by magnitude estimation and calculation skills at pre-school. Conclusion: Our results indicate that SFON first and foremost influences deeper understanding of numerical concepts at pre-school and—in contrast to previous findings –affects only indirectly children’s arithmetical development at school.

## 1. Introduction

Kindergarten children have astonishing numerical and mathematical knowledge which shows considerable interindividual differences at the end of pre-school. These differences were found to be stable and to increase over time [1]. This raises the question of why some children seem to learn numerical and mathematical principles much easier than others even without formal schooling. Previous research has identified several specific and domain-general skills influencing the acquisition of numerical and calculation skills. Children’s spontaneous and self-initiated tendencies to focus on numerical aspects in their environments (called SFON) allow them to practice their enumeration skills in everyday activities. Whilst SFON has been shown to advance numerical and mathematical competencies in kindergarten [2], its development is still not fully understood.

The main objectives of the current study were therefore to explore the development of SFON and to longitudinally examine the interrelation of SFON and later arithmetical skills. This enabled us to investigate whether SFON is linked directly to arithmetical skills at school or indirectly by facilitating numerical precursor skills, which in turn influence arithmetical skills at school.

### 1.1. Development of Numerical Skills

Research suggests that infants are able to represent numerosities using two distinct, innate representational core systems [3]: an object tracking system to exactly represent, compare, and manipulate small sets of objects (up to four) and an analog magnitude system to represent large numbers as sets. Here, successful discrimination between two quantities depends on the ratio between two numbers [4,5,6].

Several developmental models have been proposed to describe how numerical competencies are acquired during childhood [7,8,9]. These models differ in terms of the hierarchical progression of numerical competencies [10]. Dehaene’s triple-code model [11] is a neuropsychological model which describes number processing in adults and distinguishes between three number representations that are mediated by different neural networks: an analogue magnitude representation (e.g., non-symbolic comparisons), a verbal-phonological number representation (e.g., verbal counting, number fact retrieval) and a visual-Arabic number representation (e.g., solving written arithmetical problems) [12]. Von Aster and Shalev [9] extended Dehaene’s model by proposing a four-step developmental model describing how different numerical representations develop during childhood [12]. They assume that different representations of numbers are acquired in a quasi-hierarchical fashion over four steps. The basic and necessary prerequisite for numerical skills is the core system of magnitude (step 1). It refers to the implicit analog magnitude system. This innate ability forms the basis for the culturally mediated acquisition of linguistic (step 2) and visual-Arabic number symbols (step 3). A first stable numerical form of representation, i.e., a linguistic number representation (step 2), is already established in the left hemispheric language areas before school entry (and thus without systematic schooling). A second, non-linguistic form of representation is learned, i.e., the Arabic notation system (level 3) during pre-school and early primary school years. This visual-Arabic number representation with its place value and culture-specific syntax forms a network in the left and right occipito-temporal cortex areas. Linguistic and visual-Arabic number representation enable the formation of a third form of representation. The innate, core-system of magnitude (step 1) is transformed into an abstract-symbolic number line representation within the parietal cortex (step 4). Furthermore, the development of number representation modules depends on the growing capacity of non-numerical, domain-general skills (e.g., working memory and attention) [12,13].

### 1.2. Relationship between Pre-School Numerical Skills and Arithmetical Skills at School

Previous longitudinal studies highlighted the importance of early numerical skills, such as reciting the number sequence, object counting, simple calculations, subitizing and symbolic and non-symbolic comparisons for the development of arithmetical skills at school [14,15,16,17,18,19]. These skills were often summarized under different terms (e.g., number sense [20], basic quantity–number competencies [21]) showing slightly different results regarding their predictive values for subsequent arithmetical abilities at school. However, evidence implies that some aspects of numerical skills seem more relevant than others.

When dividing early numerical skills into number (including counting, number knowledge, arithmetic and number comparison) and quantity competencies (seriation, quantity comparison and length comparison), number competencies, assessed six months before school entry, were found to be the best predictor of arithmetical skills at the end of first grade, even after controlling for intelligence, whereas quantity competencies predicted arithmetical skills only indirectly [22]. Similarly, when combining non-symbolic comparisons, subitizing and flexibility in the counting sequence [23] or tasks referring to the counting sequence, counting objects, subitizing and basic arithmetic [24], these explained between 25% and 50% of variance in arithmetical skills at school. In contrast, the conceptional understanding of quantities (invariances and seriation) did not explain additional variance [24]. Von Aster et al. [25] examined different numerical skills of kindergarten children using the ZAREKI-K. Factor analysis identified four factors. These factors also differed between children with subsequent learning disorders. Based on their analyses as well as content considerations of the selected and adapted subtests of the ZAREKI-K, three factors (later referred to as domains) were defined and validated using confirmatory factor analyses. Counting maps children’s ability to recite and apply the number word series. This is conceptionally different from Number Knowledge, which summarizes the ability to grasp visual representation of numbers as well as to assign a quantity-related meaning. Magnitude Estimation/Calculation describes the ability to grasp quantities quickly and to compare them with one another. This also includes the ability to relate numbers to content, to match visually presented quantities and to perform basic arithmetic operations. Combining these two abilities was based on the results of von Aster and colleagues [26].

### 1.3. Spontaneous Focusing on Numerosity (SFON)

Research into children’s spontaneous focusing on the numerical aspects (SFON) in everyday contexts started nearly 20 years ago (for a recent review see [2]). This line of research is based on the observation that children differ in how much they pay attention to numerical aspects in settings that are not mathematical in the first place. According to Hannula and Lehtinen [27], this self-initiated attentional process serves as a trigger, because exact number recognition is not a completely automatic process. Thus, children’s SFON tendencies describe the degree of self-initiated exact number recognition without guided activation of the counting process in their natural surroundings. Hence, children with higher SFON tendencies have multiple possibilities to practice enumerating skills within everyday activities. This in turn facilitates knowledge and practice regarding numerical skills, therefore increasing the possibility to focus on numerosity in new tasks and situations. This line of argument is supported by studies revealing a link between SFON tendencies and numerical skills in pre-school children [27,28]. Previous research investigated the concurrent relationship between SFON and counting, subitizing and basic arithmetical skills in pre-school and primary school children [27,28,29,30,31,32,33,34,35]. This link could not be explained by motivational factors [29,36] or general cognitive skills [27,30,35,37]. Moreover, subsequent studies showed that SFON in pre-school is positively related to mathematical skills up to seven years later [30,36,38,39,40].

Furthermore, longitudinal studies focusing on the transition from kindergarten to school support the assumption of SFON as a direct predictor of mathematical achievement at school. For example, McMullen and colleagues [39] found in a small sample of 36 children that counting elaboration skills and SFON at the age of six were independent predictors of rational number conceptual knowledge at the age of 12. Moreover, SFON and verbal counting skills at the age of six were independent predictors of mathematical skills at the age of 12 [38]. However, after controlling for nonverbal intelligence, only SFON remained a significant predictor of mathematical skills at school.

### 1.4. Development of SFON

SFON is not innate in nature. Spontaneous numerical focusing develops over childhood [41]. Several studies revealed remarkable interindividual differences between children’s SFON tendencies, which were relatively stable over time [27,31]. While previous research focused on the role of SFON in mathematical development, we know little about what influences the development of SFON itself. Intervention studies demonstrated that the self-initiated attentional process for numerical aspects can be enhanced through guided instructions that focused on numerical activities in pre-school [42]. Moreover, integrating early numeracy interventions into daily kindergarten routines (e.g., noticing numbers and paying attention to numerical aspects) enhanced cardinality skills as well as SFON [43]. However, not only kindergarten professionals were able to foster children’s SFON. By using numerical prompts in informal play situations, parents were able to enhance children’s focusing attention on the exact number of objects [44]. As emphasized by Hannula-Sormunen and colleagues [43], SFON is a skill that can be learned. They identified shared sociocultural experiences as the main source for learning. This is of particular significance for adults involved in the mathematical education of young children, as facilitating SFON increases children’s possibilities to practice enumerating skills in their everyday activities, thus supporting their mathematical development.

However, this link between SFON and mathematical skills is not unidirectional. Studies analyzing this relationship longitudinally with repeated assessment of SFON revealed a reciprocal link: counting skills affected the development of SFON [27]. Furthermore, SFON at the age of six was found to mediate the association between subitizing at the age of five and mathematical achievement at the age of 12 [38]. In conclusion, research suggests that the elaboration of counting skills and subitizing are important for the development of SFON.

### 1.5. Domain-General Abilities as Predictors of Mathematical Skills

SFON and mathematical skills are influenced by several domain-general abilities. General intelligence proved to be an important predictor of later math achievement in children [45]. More specifically, nonverbal intelligence seems to play a crucial role in mathematical development [46] as it was found to be related to early numerical and mathematical skills in children, even before entering formal schooling [17,47,48]. The results of longitudinal studies suggest that nonverbal intelligence is directly involved in the construction of mathematical precursor skills as higher intelligence facilitates the underlying learning process [17,23]. Research also found a link between nonverbal intelligence and SFON [27,36,37,49]; for controversial results see [20].

Furthermore, research acknowledges that solving mathematical problems involves working memory [50]. Baddeley and Hitch [51] described three underlying subsystems: the central executive, the phonological loop, and the visuospatial sketchpad. Previous research showed that younger children mainly rely on resources of the visuospatial sketchpad to solve mathematical problems [52,53]. Moreover, Krajewski and Schneider [54] found a direct link between the visuospatial working memory, assessed in kindergarten, and quantity–number competencies, which in turn predicted math achievement at the end of first grade. Thus, similar to the influence of nonverbal intelligence, visuospatial working memory seems to be directly involved in the acquisition of pre-school precursor skills which then contribute significantly to the acquisition of mathematical competencies. However, due to methodological differences between studies, results are inconclusive, with some finding no link between visuospatial working memory and SFON [32,37,55], while Torbeyns and colleagues [49] did when using an odd-one-out task.

School-aged children with dyscalculia have been found to show significantly more attentional problems than their normally achieving peers [56]. Capano and Minden [57] found a prevalence of comorbid attention-deficit hyperactivity disorder and mathematics disorder of 18.1% in a sample of school-aged children. Moreover, inconsistent response times and commission errors (mistakenly marked non target letters) in a continuous attention performance test could predict arithmetic achievement [58]. In particular, visual attention in kindergarten proved to be a good predictor of later mathematical abilities up to the second grade [1,59]. Despite the definition of SFON being a self-initiated attentional process, attention (measured by teacher ratings) could not be linked to SFON [33] so far.

### 1.6. Aims of This Study

As previous research acknowledges the importance of SFON for the development of numerical and mathematical skills, the present study aimed to examine the following two issues:

1. Children show interindividual differences in SFON. However, we still do not fully understand how SFON develops and what factors may influence its development. Results of reciprocal path analyses showed that counting skills predict SFON in preschool. Furthermore, previous research revealed a link between SFON and nonverbal intelligence. Whilst working memory, attention and SFON have been examined before, it remains unclear how these skills are related to SFON. Thus, we investigated the effects of visuospatial working memory and visual attention as well as nonverbal intelligence and numerical skills.
2. The second objective was to broaden our understanding of the relationship between SFON and mathematical skills; more specifically, we aimed to examine whether SFON (1) directly influences the acquisition of calculating skills at school or (2) does so indirectly as a prerequisite of pre-school numerical skills that predict school mathematics.

Previous longitudinal studies looking at the transition from kindergarten to school support the assumption of SFON as a direct predictor of mathematical achievement at school. However, these studies only considered counting skills and subitizing as specific predictors of later school mathematics, thus potentially missing further significant numerical skills present at pre-school. To analyze these relationships in more detail, a variety of numerical skills were included in this study. Based on theoretical considerations and empirical analyses, these numerical skills were subsequently allocated to different homogeneous numerical domains: counting, number knowledge, and estimation and basic calculation skills. To further clarify the interrelations between SFON and early numerical skills at pre-school and their impacts on arithmetic at school, SFON and the three numerical domains were examined longitudinally, with two measurement points, before school entry. This is the first study to investigate the relationships between SFON and a variety of numerical skills across two time points in pre-school as well as their influence on arithmetic in school.

**Figure 1:**
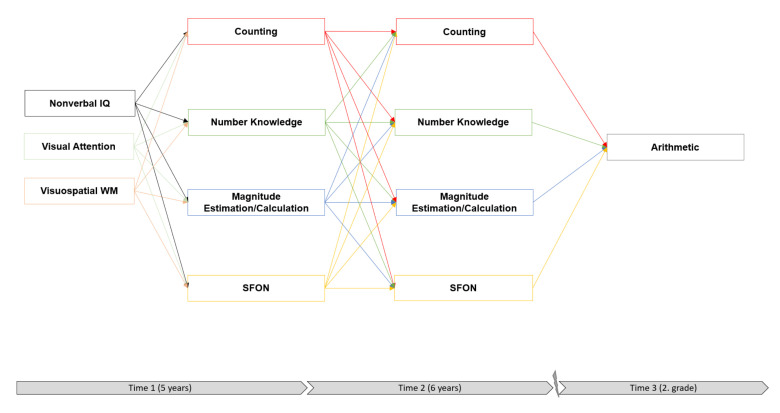
Theoretical model.

In order to answer these research questions, a theoretical model was developed based on previous research findings. In contrast to previous longitudinal studies usually conducting only one measurement point before school entry to predict later arithmetic achievement, our model enabled us to analyze the complex relationships between SFON and numerical skills at pre-school and arithmetical skills at school two years later (see Figure 1) as well as intercorrelations among variables during pre-school (Time 1 and Time 2) and indirect effects of SFON on arithmetic. Numerical skills were assumed to best predict the same skills at a later measurement point. Thus, autoregressive paths were included in order to control for stability effects of numerical skills. Because such models are highly controlled, in comparison with conventional path models without autocorrelations, no large effects can be expected [30,40].

## 2. Method

### 2.1. Participants

The current study analyzed data from the SCHUES project (“Schulbezogene UES (SCHUES)—Prävention und Therapie unter Einbezug neuronaler Korrelate und des Entwicklungsverlaufs”), a study funded by the Federal Ministry of Education and Research and approved by the local ethics committee. Overall, 1868 children (908 girls and 960 boys) attending their second of three years of kindergarten were recruited in local kindergartens in Potsdam and Berlin, Germany, by seeking parental consent before data collection. Children were tested in their second (Time 1) and final years of kindergarten, on average 9 months later (*SD* = 1.8, range: 4–15; Time 2) and again at school with an interval of *M* = 22 months (*SD* = 1.6, range: 16–28; Time 3). At Time 3, 64.1% (*n* = 1197) of the children attended the second grade, 17.5% (*n* = 140) the first grade and 0.5% (*n* = 10) the third grade. Mean ages were 63.0 months (*SD* = 4.4, range: 49–81) at Time 1, 72.42 months (*SD* = 4.2, range: 60–89) at Time 2 and 94.8 months (*SD* = 4.1, range: 79–111) at Time 3. The majority of the children (98.3%) were speaking German at home with at least one parent. A total of 520 children (27.8%) could not be examined at Time 3.

### 2.2. Materials and Procedure

Children were tested individually in a quiet room at their kindergartens in two separate sessions (Time 1 and Time 2) or at school in one session (Time 3). More specifically, at Time 1, nonverbal intelligence, visual attention and one of two SFON tasks were tested during the first session, whereas a second SFON task, visuospatial working memory and mathematical abilities were administered at the second session. At Time 2, one of two SFON tasks was followed by tasks assessing numerical competencies in two separate sessions. At Time 3, arithmetic was tested in one session. All experimenters received comprehensive training. Their training included a two-day workshop conducted by senior project members and a videotaped trial testing session with a child which was evaluated by project members as well as one supervised testing session at the kindergarten or school within their first week of testing.

#### 2.2.1. SFON

At Time 1 and Time 2, two SFON tasks were administered. Before and during the SFON tasks no mathematical or quantitative comments were made, no mathematical task was done before the task and no feedback was given during the task in order to avoid suggesting the mathematical nature of SFON tasks. During the tasks, children’s comments were recorded. Children were classified as focusing on numerosity if they (a) produced the correct number, (b) mentioned number words referring to the task (e.g., ‘I scooped three times’), (c) asked for the correct number (e.g., ‘How many candies did you give the bird?’) or (d) used at least one of the following counting acts: whispering numbers, indicating counting acts by fingers, head nodding, lip movements or counting with fingers. The maximum score on every SFON task was 4. The two SFON tasks correlated moderately at both time points: Time 1: *r* = 0.37 (*p* < 0.001, *n* = 1812); Time 2: *r* = 0.43 (*p* < 0.001, *n* = 1651). The scores of the two SFON tasks for Time 1 and Time 2 were added, resulting in a maximum score of 8. Chronbach’s Alpha was 0.80 (*n* = 1812) for Time 1 and 0.83 (*n* = 1651) for Time 2.

##### SFON Imitation Task with Two Numerosities (Bird at Time 1 and Postbox at Time 2)

The materials for Time 1 were a toy parrot and two bowls of coloured glass pebbles, that were placed in front of the child (for a more detailed description, see [27,30]). The experimenter sitting next to the child introduced the task by pointing and saying: ‘This is the bird Elsi. It likes candies. There are red candies here and blue candies here. Please watch carefully what I do, and then you do just like I did, okay?’. For the first trial, the experimenter lifted two red candies and one blue candy into the bird’s mouth one at a time using a large hand movement. The candies dropped into the bird’s stomach, making a bumping sound. Then, the child was told: ‘Done; now you do exactly what I did.’ For the second trial three green and two white candies were used, for the third trial two transparent and three black candies were used, and for the fourth trial one yellow and two light blue candies were used. The materials for Time 2 were a postbox and two stacks of different coloured envelopes placed in front of the child (for a more detailed description, see [27]). The instructions and numbers of envelops posted into the postbox were identical to the bird task.

##### SFON Imitation Task with One Numerosity (Truck at Time 1 and Chicken at Time 2)

**Figure 2:**
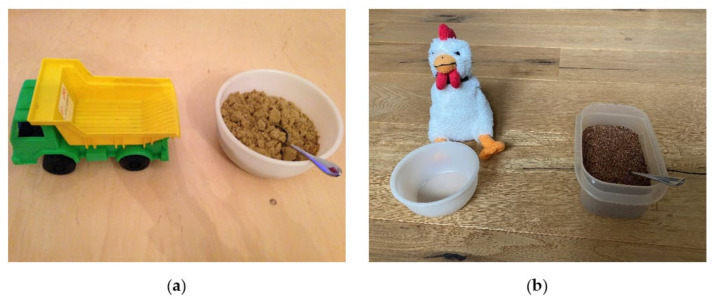
Pictures of the experimental setting of the SFON tasks with one numerosity: (**a**) truck task at Time 1; (**b**) chicken task at Time 2.

As there are no details published about SFON imitation tasks with only one numerosity, these are described in more detail here. The materials for Time 1 were a toy truck, a bowl with coarse sand and a spoon placed in front of the child (see Figure 2) [42]. The experimenter sitting next to the child introduced the task by pointing to the materials and saying: ‘This is a truck, and this is gravel. Watch carefully; I scoop gravel onto the truck and then you scoop gravel onto the truck, okay?’. In the first trial, the experimenter scooped twice using a large hand movement. Then the child was told: ‘Now, you scoop gravel onto the truck.’ The number of scoop movements was as follows: for the second trial, three times, for the third trial, two times and for the fourth trial, one time. The materials for Time 2 were a toy chicken with a bowl, a bowl with grain and a spoon. The instructions and the number of scoops were identical to the truck task.

The instructions of the SFON task with one numerosity and the SFON task with two numerosities were not identical (two numerosities: ‘Now, you do exactly like I did” vs. one numerosity: ‘Now, you shovel gravel on the truck.”). In a sub-sample of 102 four- to five-year-old children, we examined whether instructions affected SFON tendencies. Half of the children were presented the two SFON tasks of Time 1 using the instructions as described above. The other half were presented the two SFON tasks with interchanged instructions. During the Elsi task children were instructed with the following words: ‘Now it’s your turn’. In the truck task, the children were told: ‘Now, you do exactly like I did’. Groups were matched regarding age, sex and their subtest score in the “Understanding Number and Quantity” section of the BUEVA-III [60]. Within SFON tasks (Elsi: *z* = −0.69, *p* = 0.490 and truck: *z* = −1.16, *p* = 0.110) there were no differences regarding SFON tendency between the two different instructions.

#### 2.2.2. Early Numerical Skills

Early numerical skills at Time 1 and Time 2 were assessed using a modified version of the neuropsychological test battery ZAREKI-K [26]. The three math-related domains Counting, Number Knowledge and Magnitude Estimation/Calculation were derived from two confirmational factor analyses at Time 1 (*χ²* (62) = 308.04, *p* < 0.001, RMSEA = 0.05, CFI = 0.97, *n* = 1828) and Time 2 (χ² (62) = 215.46, *p* < 0.001, RMSEA = 0.04, CFI = 0.96, *n* = 1714). The task descriptions of the early numerical skills at Time 1 and Time 2 are summarized in Table 1.

#### 2.2.3. Arithmetic

Mathematical performance at school (Time 3) was assessed using the subtests addition, subtraction, division, halving and doubling of the grade specific standardized German mathematics test DEMAT 2+ [61]. The reported Chronbach’s Alpha of subtests included in this study ranged from 0.68 (subtraction) to 0.87 (doubling). Children attending first grade completed the DEMAT 1+ [62]. Here, the reported Chronbach’s Alpha was 0.89. For third grade children, the subtest arithmetic of the DEMAT 3+ [63] was used. The reported Chronbach’s Alpha for arithmetic was 0.80. DEMAT tests are based on curricula representative of the German federal states and assess school-related math performance. For every version of the DEMAT, the results of the administered subtests were added and standardized in half year age intervals. The respective sample sizes for standardization includes all children attending the respective school year (DEMAT 1+: *n* = 1119, March–September 2013; DEMAT 2+: *n* = 1021, February–September 2014; DEMAT 3+: *n* = 1148, February–November 2015).

#### 2.2.4. Nonverbal Intelligence

The subtest Nonverbal Intelligence of the BUEVA-III [60] was used to assess the nonverbal intelligence at Time 1. BUEVA-III is a German test battery for assessing children’s stages of development and to identify children at risk for later developmental disorders before school entry. The subtest Nonverbal Intelligence assesses children’s abilities of logical thinking and reasoning by analogy. Children have to identify the picture out of a set of pictures which does not belong with the others (Chronbach’s Alpha is 0.87 [60]). Children’s performance in the Nonverbal Intelligence subtest has been shown to correlate *r* = 0.54 (*p* < 0.001) with their performance in the Colored Progressive Matrices [64].

#### 2.2.5. Visuospatial Working Memory

A Corsi Block task [65] was used to assess visuospatial working memory. Behavioral and neuropsychological evidence demonstrated correspondence between spatial short-term memory and performance in the Corsi Block task (for a review, see [66]). Former studies demonstrated the applicability of the Corsi Block task for children aged four or older [53,67,68,69,70]. The material consists of a wooden board with six red wooden blocks nailed to it in a random order [69,70]. The experimenter pointed at a sequence of blocks starting from a span of two and instructed the child to point to the same blocks in the same order. For each span, the experimenter showed two simple and two complex sequences before progressing to three blocks. The maximum span was five blocks. The task was terminated after the child failed four consecutive trials. Children received one point for a correct answer. The maximum score was 16 points. Chronbach’s alpha was 0.83 (*n* = 1820). Schmid et al. [69] found a retest reliability (three weeks) of *r_tt_* = 0.61. After exclusion of processing speed, the Corsi Block task was found to correlate with Matrices (*r* = 0.50, *p* < 0.01) and hand movements (*r* = 0.72, *p* < 0.01), but not with the phonological loop [69].

#### 2.2.6. Visual Attention

Visual Attention was assessed by the subtest Attention of the BUEVA-III [60], which aims to evaluate children’s abilities to maintain visual attention by assessing the speed and accuracy with which they can identify two target pictures from a range of different pictures. Children were asked to go through the pictures, line by line, and mark every picture that depicted the target images (dog or elephant). The test was terminated after 90 s. A score for Visual Attention was computed by subtracting the total number of wrong answers from the number of correct answers. Split-half reliability in the standardization sample was 0.88 [60]. The subset Attention of BUEVA-III correlated with Attention of BUEVA-II [71] (*r* = 0.66, *p* ≤ 0.001).

### 2.3. Data Analysis

For analysis, standardized T-scores were used. To this end, the three numerical domains at kindergarten, SFON, and Visuospatial Working Memory were standardized and transferred into T-scores. T-scores of the three numerical domains in kindergarten, Nonverbal Intelligence and Visual Attention, as well as SFON, are based on half year age intervals, therefore considering children’s ages at assessment. The T-scores of Arithmetic are based on school term intervals.

Correlational analyses were conducted using SPSS version 28. Path analysis with manifest variables was used to analyze our theoretical model. This was done using the full maximum likelihood method (FIML) in MPLUS 7.1 [72]. FIML estimates missing values directly without imputing them for each individual parameter [73]. The basic model, as described in detail before, was analyzed, and all relevant parameters were estimated. Non-significant paths were removed step by step.

## 3. Results

Mean scores, standard deviations and range for performance of each tested variable are presented in Table 2 for Time 1, Time 2 and Time 3 separately, alongside descriptions for standardized T-scores. Skewness of numerical domains at Time 2 were slightly lower than at Time 1, thus reflecting a lower level of difficulty at Time 2. All skewness scores were within an acceptable range [74]. To examine development of SFON during pre-school, SFON raw scores were analyzed. SFON tendency increased over time, as children’s SFON scores at Time 1 were significantly lower compared to Time 2 (*t* (1217) = −20.01, *p* < 0.001, *r* = 0.25). The percentage of pre-school children achieving a maximum SFON score nearly tripled over time (Time 1: 9.6% vs. Time 2: 28.3%).

### 3.1. Correlational Analyses

Table 3 presents Pearson’s correlations between variables.

#### 3.1.1. Stability of Constructs

Results showed the following patterns of stability during pre-school (from Time 1 to Time 2): Number Knowledge showed medium stability that was significantly lower compared to Counting and Magnitude Estimation/Calculation, which demonstrated high stability (*p* < 0.05). Furthermore, SFON demonstrated medium stability from Time 1 to Time 2.

#### 3.1.2. Intercorrelations

Domain-general cognitive abilities showed medium intercorrelations. However, intercorrelations between numerical competencies at Time 1 and Time 2 were high. Here, Counting and Magnitude Estimation/Calculation had the highest correlation (differences to other correlational coefficients *p* < 0.10). Additionally, numerical domains showed medium to high correlations across Time 1 and Time 2, except for Number Knowledge at Time 2, which showed a lower correlation to Counting and Magnitude Estimation/Calculation at Time 2.

SFON showed significant but modest associations with numerical domains. Irrespective of simultaneous or repeated testing during pre-school, SFON and Magnitude Estimation/Calculation had the highest and SFON and Number Knowledge the lowest correlation. All correlational coefficients differed significantly from each other (*p* < 0.05). 

Numerical domains assessed at Time 1 and Time 2 had positive correlations with Arithmetic at second grade. Here, Magnitude Estimation/Calculation (Time 1 and Time 2) showed the highest correlation with Arithmetic at Time 3 (*p* < 0.05).

### 3.2. Path Analysis

Estimates of the final path model are summarized in Table 4. Here, we analyzed the proposed theoretical model from pre-school to second grade. The model fitted the data well (*χ²* (15) = 13.87, *p* = 0.535, CFI = 1.00, RMSEA < 0.001, SRMR = 0.008).

#### 3.2.1. Time 1

Nonverbal Intelligence, Visual Attention and Visuospatial Working Memory were considerably predictive of numerical domains and SFON at Time 1. Domain-general cognitive abilities explained up to 41% of the variances of numerical competencies compared to only 10% of variances in SFON. Overall, the lowest path coefficient was found for Visual Attention.

At Time 1, residuals of numerical domains intercorrelated significantly (*r* = 0.50–0.44). Regarding SFON, the highest intercorrelation with the numerical domains was found for Magnitude Estimation/Calculation (*r =* 0.13), the lowest was for Number Knowledge (*r =* 0.09).

#### 3.2.2. Time 2

For numerical domains and SFON, expected autocorrelative paths between Time 1 and Time 2 displayed medium effect sizes.

At Time 2, 56% of the variance of Magnitude Estimation/Calculation was explained by the model. In addition to numerical domains at Time 1, SFON, Nonverbal Intelligence, Visual Attention and Visuospatial Working Memory were found to be significant predictors of Magnitude Estimation/Calculation at Time 2. Furthermore, the model explained 47% of the variance of Counting at Time 2. Numerical domains, SFON (marginal), Visual Attention and Nonverbal Intelligence were significant predictors of Counting at Time 2. Regarding Number Knowledge at Time 2, only numerical domains and Nonverbal Intelligence at Time 1 showed significant paths and explained, in total, 29% of the variance.

For SFON, the model explained 23% of the variance at Time 2. Counting, Magnitude Estimation/Calculation (but not Number Knowledge) and Visual Attention showed significant path coefficients.

At Time 2, residuals of numerical domains intercorrelated at a low level (*r =* 0.17–0.24). Residuals of SFON only correlated with residuals of Magnitude Estimation/Calculation.

#### 3.2.3. Time 3

Arithmetic at second grade was predicted mainly by performance in Magnitude Estimation/Calculation at both time points. Only Number Knowledge (not Counting) at Time 2 added some additional explanation of variance at a very low level. SFON could not predict later arithmetical skills directly. Thus, using a mediation model, we tested whether SFON at Time 1 was associated with Arithmetic at Time 3 via Magnitude Estimation/Calculation at Time 2. A small but significant indirect path was found (*B* = 0.01, *SE* = 0.01, *β* = 0.01, *p* = 0.040).

## 4. Discussion

In a large epidemiological sample, several different numerical skills, SFON and general cognitive performance were repeatedly assessed during pre-school along with arithmetical skills at second grade. This enabled us to analyze the complex relationships between SFON, numerical skills and arithmetical skills during the course of childhood development as well as intercorrelations among variables during pre-school (Time 1 and Time 2) and indirect effects of SFON on arithmetical skills. Numerical domains as well as SFON at pre-school were expected to influence arithmetical skills at school. Moreover, by including domain-general cognitive abilities into our longitudinal model, we were able to examine their contributions to the development of SFON before school entry.

### 4.1. The Development of SFON

In line with previous research suggesting that SFON is not an innate ability, but rather develops over childhood [41], our results found a significant increase in SFON tendencies over a nine-month time period at pre-school with more than three times as many children achieving a maximum SFON-score at Time 2.

Domain-general cognitive abilities (i.e., Nonverbal Intelligence, Visuospatial Working Memory and Visual Attention) explained a small but significant amount of children’s SFON tendencies. While previous studies reported a link between SFON and nonverbal intelligence, neither visuospatial working memory nor attention were found to be linked to SFON. This may be due to the small sample sizes of previous studies. By contrast, a large sample, as examined our study, facilitates detection of small effects. For example, when assessing visuospatial working memory in a sample of 76 primary school children, Kucian and colleagues [32] used a Corsi Block-tapping task similar to the task used in our study where item difficulty was adapted for pre-school children (i.e., spans with lower numbers of blocks). They found a positive but non-significant correlation between SFON and visuospatial memory (*r* = 0.20), which did not differ significantly from the correlational coefficient of *r* = 0.24 at Time 1 found in this study (*z* = 0.35, *p* = 0.364).

Another reason for contrasting findings in previous studies may be due to different methods used to assess SFON and domain-general cognitive abilities. Different types of tasks have been designed for assessing SFON. All SFON tasks avoid mathematical references before as well as during the tasks, include only small numbers of objects and are designed to avoid or minimize influences of working memory capacity, visuo-motor and comprehension skills [27]. To our knowledge, measuring the development of children’s SFON tendencies and their interrelations with numerical competencies mainly relies on the use of two types of SFON tasks: picture tasks and action-based tasks. Our study used the later: children were instructed to imitate the action of the experimenter. In contrast, during picture tasks, children are shown pictures with objects in varying numbers and are asked to describe the pictures they have seen [55]. Both types of tasks demonstrated methodological advantages and limitations and were not significantly correlated [55]. Similarly, Gloor and colleagues [37] found a significant but small correlation of *r* = 0.15 between SFON and working memory in a sample of 1279 first graders, when using a working memory score (i.e., summarizing children’s results in Corsi Block tests and number sequence backwards tests) alongside a picture task to assess SFON. Within their structural equation model only nonverbal intelligence (not working memory) was able to predict SFON. Overall, these differences highlight the role of domain-general cognitive abilities when solving conceptually different SFON tasks due to different task demands. It seems reasonable to assume that children will rely more on visuospatial working memory capacity when solving action-based SFON tasks compared to picture tasks.

In line with Hannula et al. [27], we found a reciprocal path between Counting and SFON. Furthermore, by including Magnitude Estimation and Calculation, we were able to demonstrate their influence on the development of SFON. Notably, Number Knowledge seemed to play no role at all. Higher skills in magnitude estimation and basic calculation seem to increase the probability of focusing on numerosity in new tasks and situations. Hence, children’s skills in magnitude estimation and calculation were found to be not only of crucial importance for later arithmetical skills at school, but also to foster children’s SFON tendencies. Interestingly, SFON and number knowledge seem to be two distinct number-processing skills, which were not associated at pre-school, and both variables explained only a small percentage of variance in our model. This suggests that children’s development of SFON and number knowledge depend on additional factors not examined in this study.

However, this study was the first to examine the development of SFON and demonstrated that children’s SFON tendencies increase remarkably during pre-school. Furthermore, domain-general cognitive abilities (nonverbal intelligence, visual attention, visuospatial working memory) as well as domain-specific numerical skills (counting, magnitude estimation, calculation) were able to explain to some degree differences found in children’s SFON tendencies. However, future research examining the possible influences of additional variables, such as inhibitory control, is needed to explain the development of SFON and its relationship with numerical competencies. Inhibitory control, a subcomponent of the central executive, is the ability to suppress irrelevant or misleading information [75]. Previous studies have shown that inhibitory control is associated with mathematical competencies [76,77]. In order to practice mathematical skills by paying attention to relevant numerical aspects in everyday contexts, children may benefit from suppressing concurrent information.

### 4.2. Interrelations between SFON and Early Numerical Competencies

Correlations between SFON and numerical competencies were small to moderate across measurement points. Furthermore, SFON explained only a very small amount of variance of Counting and Magnitude Estimation/Calculation while no influence on Number Knowledge was found. A closer look at previous studies using imitation tasks to assess SFON revealed correlational coefficients of similar size [27,28,30,34,40]. However, a significantly higher correlation (*r* = 0.38) was found in a sample of 355 Ecuadorian five-to-six-year-olds when using an imitation task (*p* < 0.05 for all comparisons of Time 1 and Time 2 except Magnitude Estimation/Calculation of Time 2 with *p* = 0.067 [49]). Thus, SFON seems more relevant to the development of numerical competencies referring to the estimation of magnitudes and basic calculations than to counting. When analyzing pre-school children’s math abilities and SFON profiles, Gray and Reeve [78] found high-SFON children were more likely to belong to the “strong math, poor count sequence” math ability profile. Children who fell into this category performed well in math tests, including number naming, while under-performing in counting tests (counting sequence and object counting). While this is not in line with our finding where SFON showed the lowest association to Number Knowledge, results of both studies imply that SFON may be more relevant for developing a deeper understanding of mathematical concepts than for learning procedural routines like reading number words or reciting the counting sequence. This evidence is crucial for a better understanding of the SFON concept itself. All known SFON tasks use non-symbolic numerical representations. However, Rathé and colleagues [79] developed a novel concept called SFONS (Spontaneous focusing on Arabic number symbols). SFONS tendencies reflect children’s spontaneous attention to Arabic number symbols in their everyday surroundings. A higher SFONS tendency is assumed to enhance children’s mathematical development by providing them with more self-initiated opportunities for practicing Arabic numeral knowledge. The authors found that the relationship between SFONS and Arabic numeral identification (“What number is this?”) differed between age-groups. While there was a strong relationship between SFONS (but not SFON) and Arabic numeral identification in three-year-olds (*n* = 30), there was no significant association between SFONS and Arabic numeral identification, verbal counting or counting objects in five-year-olds (*n* = 36). These results are interesting and warrant further investigation, as they may help to explain the missing association between SFON and Number Knowledge in our study of children aged five and six years.

### 4.3. SFON as an Indirect Predictor of Later Arithmetical Skills

We did not find a direct path from SFON at pre-school to arithmetic at school, as this association was fully mediated by the numerical domain of Magnitude Estimation/Calculation. Furthermore, children’s Counting skills at pre-school did not add additional explanation of variances. Only children’s Number Knowledge directly predicted Arithmetic at school, albeit with a small effect size range. However, compared to conventional path models without autocorrelations, we did not expect large effects, as our model was highly controlled due to the autoregressive paths of numerical skills in pre-school. In addition, the skills of the domain Magnitude Estimation/Calculation also included basic arithmetical skills.

The results of our study show that at kindergarten—without formal instruction of mathematics—there already are interindividual differences regarding magnitude estimation and calculation, which seem to further influence the development of subsequent arithmetical skills at school. Both SFON and counting skills independently support the development of these early mathematical competencies before school entry. Thus, it seems reasonable to assume that SFON is relevant for developing a deeper understanding of mathematical concepts in pre-school, which in turn are important for higher arithmetical skills at school. Higher SFON tendency may serve as a developmental advantage, as it supports the development of relevant precursor skills. In line with this, Lepola and Hannula-Sormunen [36] found children’s SFON tendencies (assessed using action-based tasks) at the end of kindergarten directly contributed to basic arithmetical skills one year later, at grade one, and indirectly predicted advanced arithmetical skills at grade two.

### 4.4. Limitations

At pre-school, numerical and calculation tests were used, which differentiated within the low achievement range as the SCHUES project aimed to detect children at risk for later math difficulties. This is reflected by the negative skewness of the two domains Counting and Number Knowledge found at Time 1 and Time 2, thus resulting in lower stability rates and paths coefficients between numerical and calculation competencies and arithmetical skills at school. However, there is no reason to assume that interrelations between SFON and numerical competencies were affected to a substantial degree.

Additionally, using path modelling to examine the longitudinal relationships of our variables did not control for measurement errors in the assessment by modelling latent variables. Lastly, we only examined the development of SFON over the last year at kindergarten. As previous studies have also found interindividual SFON differences in younger children [27] future research should expand the age range in order to further investigate the developmental trajectory of SFON.

### 4.5. Practical Implications

To summarize, SFON develops significantly over the last year of kindergarten and seems to moderately influence mathematical development before school. Higher SFON tendencies seem to facilitate a deeper understanding of mathematical skills at pre-school. However, effect sizes were small and our results do not support the assumption that SFON directly influences the development of school arithmetic. The influence of SFON on children’s numerical development seems to be limited to the development of numerical skills before formal instruction. The previously found link between SFON and mathematics at school seems to be of an indirect nature.

Furthermore, our results showed that skills belonging to the domain of Magnitude Estimation/Calculation were the most important predictors of arithmetic at school. This has implications for mathematical education at pre-school. Similarly, the importance of fostering mathematical skills before school entry had been shown previously [80,81]. Here, structured training programs especially designed to support children at risk for school-related math difficulties were assessed. Enhancing children’s attention towards numerical aspects in their natural environment has been shown to support not only SFON itself but numerical skills as well [42,43,44]. Thus, it offers practical and low-threshold possibilities for pre-school teachers and parents to support children’s mathematical development.

## Figures and Tables

**Table 1:** Descriptions of numerical and basic arithmetical subtests at Time 1 and Time 2.

Subtest	Description	Number of Items	Maximum Score
*Counting ^1^*			12
Counting forward	to count forward as far as possible (one point for correctly counting to 5)	stopping at 22	5
Counting backwards	to count backwards from 9 to 1	1	4
Counting objects	to count beetles pictured on paper (7, 5 and 14 beetles)	3	3
*Number Knowledge ^2^*			12
Number reading	to read aloud numbers in their Arabic form (3, 5, 4, 7)	4	4
Number–object correspondence	to identify which picture of objects matches the Arabic number (3, 5, 4, 7)	4	4
Object–number correspondence	to identify which Arabic number matches the display of dots (4, 2, 5, 6)	4	4
*Magnitude Estimation/Calculation ^3^*			Time 1: 35, Time 2: 39
Basic visual arithmetic	to solve visually presented addition and subtraction problems by using wooden cubes: how many cubes must be added/removed to have the same amount	6	12
Basic verbal arithmetic	to solve addition and subtraction problems presented verbally	Time 1: 6, Time 2: 8	Time 1: 6, Time 2: 8
Subitizing	to estimate how many dots had been presented during 1 s (3, 2, 4)	3	3
Magnitude comparison	to identify which set of dots is larger in quantity when presented for 1 or 5 s	Time 1: 10, Time 2: 12	Time 1: 10, Time 2: 12
Quality estimation of quantities in context	to estimate whether some quantities in context (spoken sentences) seem to be few, normal, or many (i. e., ‘30 cuddly toys in a bed. Is this few, normal or many?’)	4	4

Cronbach’s alpha (*n* = 1828 at Time 1; *n* = 1714 at Time 2): ^1^ Time 1 = 0.60, Time 2 = 0.54; ^2^ Time 1 = 0.90, Time 2 = 0.82; ^3^ Time 1 = 0.86, Time 2 = 0.83.

**Table 2:** Descriptive statistics.

		*Raw Scores*			*Standardized Values*			
	*n*	M	SD	Range	M	SD	Range	Skewness
*Time 1*								
Counting	1824	8.17	3.05	0–12	49.76	9.41	21–67	−0.23
Number Knowledge	1821	8.86	3.48	0–12	49.70	9.16	24–64	−0.39
Estimation/Calculation	1812	26.48	5.75	6–35	49.86	9.92	21–76	−0.03
SFON	1812	3.81	2.55	0–8	50.00	9.30	35–69	0.06
Nonverbal Intelligence	1868	23.36	5.00	0–33	48.34	9.07	20–60	−0.61
Visual Attention	1856	17.35	14.40	−90–51	48.19	9.13	20–60	−0.56
Visuospatial WM	1821	7.24	3.53	0–16	49.75	9.88	21–81	0.05
*Time 2*								
Counting	1657	10.44	2.14	0–12	49.00	8.69	19–60	−0.84
Number Knowledge	1652	10.98	1.81	0–12	48.79	9.26	19–59	−1.14
Estimation/Calculation	1651	32.59	4.49	11–39	49.30	10.28	20–78	−0.53
SFON	1651	5.36	2.53	0–8	49.96	9.07	31–64	−0.29
*Time 3*								
Arithmetic (DEMAT) *^1^*	1347	-	-	-	49.69	9.87	17–77	0.06

^1^ Because of structural differences, raw scores for DEMAT vary for each DEMAT version, thus were not reported here.

**Table 3:** Correlations.

Variable	1	2	3	4	5	6	7	8	9	10	11
*Time 1*											
1. Counting											
2. Number Knowledge	0.60										
3. Estimation/Calculation	0.63	0.58									
4. SFON	0.23	0.21	0.29								
5. Nonverbal Intelligence	0.38	0.37	0.52	0.24							
6. Visual Attention	0.35	0.34	0.43	0.21	0.43						
7. Visuospatial WM	0.37	0.29	0.44	0.23	0.40	0.31					
*Time 2*											
8. Counting	0.65	0.53	0.51	0.21	0.35	0.31	0.29				
9. Number Knowledge	0.42	0.49	0.40	0.15	0.28	0.21	0.19	0.49			
10. Estimation/Calculation	0.58	0.54	0.68	0.26	0.48	0.40	0.38	0.54	0.48		
11. SFON	0.26	0.23	0.32	0.41	0.23	0.26	0.21	0.24	0.19	0.32	
*Time 3*											
12. Arithmetic (DEMAT)	0.40	0.37	0.49	0.14	0.29	0.24	0.26	0.35	0.31	0.52	0.18

For all coefficients *p* < 0.001.

**Table 4:** Path analysis results.

	*B*	*SE B*	*β*	*B*	*SE B*	*β*	*B*	*SE B*	*β*	*B*	*SE B*	*β*
	*Time 1*											
*Time 1*	**Counting**			**Number Knowledge**			**Estimation/Calculation**			**SFON**		
	***R²* = 0.24 **			***R²* = 0.21 **			***R²* = 0.41 **			***R²* = 0.10 **		
Nonverbal Intelligence	0.23	0.02	0.22 ***	0.25	0.02	0.24 ***	0.41	0.02	0.37 ***	0.15	0.03	0.14 ***
Visual Attention	0.19	0.02	0.18 ***	0.19	0.02	0.18 ***	0.23	0.02	0.21 ***	0.11	0.03	0.11 ***
Visuospatial WM	0.23	0.02	0.24 ***	0.15	0.02	0.17 ***	0.27	0.02	0.27 ***	0.15	0.02	0.16 ***
	***Time 2***											
*Time 1*	**Counting**			**Number Knowledge**			**Estimation/Calculation**			**SFON**		
	***R²* = 0.47 **			***R²* = 0.29 **			***R²* = 0.56 **			***R²* = 0.23 **		
Counting	0.42	0.02	0.45 ***	0.13	0.03	0.13 ***	0.19	0.03	0.17 ***	0.06	0.03	0.06 *
Number Knowledge	0.18	0.02	0.19 ***	0.35	0.03	0.34 ***	0.17	0.03	0.15 ***	--	--	--
Estimation/Calculation	0.07	0.02	0.08 ***	0.10	0.03	0.10 ***	0.39	0.03	0.37 ***	0.17	0.02	0.18 ***
SFON	--	--	--	--	--	--	0.04	0.02	0.04 *	0.33	0.02	0.34 ***
Nonverbal Intelligence	0.05	0.02	0.05 *	0.06	0.03	0.06 *	0.14	0.02	0.12 **	--	--	--
Visual Attention	0.04	0.02	0.05 *	--	--	--	0.07	0.02	0.06 ***	0.11	0.02	0.11 ***
Visuospatial WM	--	--	--	--	--	--	0.05	0.02	0.05 **	--	--	--
	***Time 3***											
*Time 1*	**Arithmetic**											
	***R²* = 0.36 **											
Estimation/Calculation	0.10	0.03	0.28 ***									
*Time 2*												
Number Knowledge	0.10	0.03	0.09 ***									
Estimation/Calculation	0.30	0.03	0.31 ***									

*** *p* ≤ 0.001, ** *p* ≤ 0.01, * *p* < 0.05; significant (*p* ≤ 0.001) intercorrelations of residuals at Time 1: Counting and Number Knowledge: *r* = 0.50, Counting and Estimation/Calculation: *r* = 0.49, Counting and SFON: *r* = 0.10, Number Knowledge and Estimation/Calculation: *r* = 0.44, Number Knowledge and SFON: *r* = 0.09, Estimation/Calculation and SFON: *r* = 0.14. At Time 2: Counting and Number Knowledge: *r* = 0.24, Counting and Estimation/Calculation: *r* = 0.17, Number Knowledge and Estimation/Calculation: *r* = 0.22, Estimation /Calculation and SFON: *r* = 0.08.

## Data Availability

The data presented in this study are available on request from the corresponding author.
